# 

**Published:** 1898-04-02

**Authors:** 


### The Hospital

A JOURNAL OF
THE MEDICAL SCIENCES AND HOSPITAL ADMINISTRATION,
INCLUDING
A SPECIAL SECTION FOR NURSES.

EDITED BY
SIR HENRY BURDETT, K.C.B.,
AND
SOLOMON C. SMITH, M.D.

IN TWO VOLUMES ANNUALLY.

VOLUME XXIV.
April, 1898, to September, 1898.

SURGEON GENL'S OFFICE
165461.
LIBRARY.

London:
PUBLISHED BY THE SCIENTIFIC PRESS (LIMITED), AT THE HOSPITAL BUILDING,
28 & 29, SOUTHAMPTON STREET, STRAND, W.C.

MDCCCXCVIII.

W1 H069X

### INDEX TO VOL. XXIV., 1898.

*** Articles under the general heading *Medical Progress and Hospital Clinics* are distinguished by the initial (C.) for *Hospital Clinics*, and by (P.) for the rest of the series. (W.) distinguishes the *Institutional Workshop* articles.

Abdomen, Contusions of the (P.), 206
Abdomen, Gunshot Wounds of the (C.), 285
Abdomen, A Three-mile Walk with a Penetrating Wound of the, 9; *and see p.* 52
Abdominal Cicatrix, Hernia of the (P.), 206
Abdominal Incision, The (P.), 205
Abdominal Myomectomy (P.), 339
Abortifacient, Lead as an (C.), 321
Abortion, Criminal (C.), 268
"Accouchement forcé," 75
Actinomycosis, Eucalyptus in (C.), 169
Addenbrooke's Hospital, Cambridge (W.), 276
Advertisements and Ethics, 163
Albuminuria (P.), 306
Albumose (P.), 307
Alcaptonuria (P.), 307
Alien Immigration, 23
Ambulance Service in the U.S. Army (W.), 448
Amenorrhœa with Gross Lesion, 26
Anæsthetics, Administration of, to Children 369
Anæsthetics, Progress in (P.), 444
Anarchism and Madness, 421
Anatomy (Neurology) (P.), 327
Anderson, Mrs. Garrett, and the Midwives, 92
Angina Pectoris (P.), 113
Antidotal Treatment of Disease, The, 301
Antiphthisin (P.), 114
Antiseptic Precautions (P.), 149
Antiseptics (P.), 116, 412
Anti-toxin and Post-diphtherial Paralysis, 408
Anti-toxin, Diphtheria (C.), 184
Anus, Rectum and (P.), 324
Anytoles (C.), 211
Aorta, Intra-abdominal Compression of the (P.), 221
Aphasia and Testamentary Capacity, 441
Apoplexy, Trephining for (C.), 170
Appendicitis (P.), 29
Arbroath Infirmary (W.), 417
Argentamin in Conjunctivitis (P.), 309
Argentamine (P.), 12
Army Medical Department, The, 2
Arterio-Sclerosis, Cardiac Hypertrophy in, 428
Arterio-Sclerosis, Heart Failure of (P.), 113
Arthritis, Rheumatoid (P.), 78
Asepsis (Ophthalmology) (P.), 12
Asepsis in Operations in Private Practice, Methods of obtaining (C.), 334, 351
Asthma (P.), 202
Ataxy, Acute, of one Limb (P.), 271
Australia, Notes from (W.), 432
Baby-Farming, 365
Bacteriological Methods of Diagnosis in Private Practice, Notes on the Use of (C.), 424, 440
Bacteriological Laboratory, A Public, 435
Bacteriuria (P.), 307
Bad Nauheim, 331
Barnsley, Small-pox Hospital for (W.), 342
Bath Houses, German Hospital (W), 359
Bedford County Hospital (W.), 414
Best in the World, The, 230
Bicycle in Surgery, The (C.), 112
Bile Duct, Cystic Dilatation of the Common, 286
Bladder (P.), 309
Bladder, Extra-Peritoneal Rupture of (P.), 45
Bladder, Tuberculosis of the (P.), 372
Bladder, Tuberculous Disease of the (C.), 130
Board Schools, Classification in (W.), 312
Book World of Medicine, The (*Books, Magazines, and Pamphlets Reviewed*):—
Abbott. Stammering, Stuttering, and Other Speech Affections, 97
Allbutt. A System of Medicine, by Many Writers, 225, 259
Balfour. Lectures on Diseases of the Heart and Aorta, 275
Bannatyne. Rheumatoid Arthritis, 327
Beevor, Diseases of the Nervous System, 14
Bibby. The Building of Workhouses, 341
Bottone. Radiography, 225
Bradshaw's Dictionary of Bathing Places, 1898, 447
Burdett's Hospitals and Charities, 241.
Burrell, Councilman, and Folsom. Medical and Surgical Reports of the Boston City Hospital, 241
Campbell. Respiratory Exercises in the Treatment of Disease, 153
Choksy. Report on Bubonic Plague, 189
Colton. Physiology, 393
Cooke. Tablets of Anatomy, 275
Cuff. Lectures on Medicine to Nurses, 341
Dakin. Handbook of Midwifery, 117
Dent. How to Select a Life Office, 291
Dictionary of Diseases and Their Treatment, 447
Diseases and Remedies, 413
Edwards. Medicated Wines and Their Dangers, 225
Furbringer, trans. by Gilbert. Text-book of Diseases of the Kidneys and Genito-Urinary Organs, 341
Book World of Medicine (*continued*).
Gadd. A Synopsis of the British Pharmacopœia, 1898, 153
Gersuny, trans. by Levetus. Doctor and Patient, Hints to Both, 97
Giles. Physiology of Pregnancy and Childbirth, 33
Greene. Lippincott's Pocket Medical Dictionary, 393
Gygax and Landolt, trans. by Neyman. Vade Mecum of Ophthalmological Therapeutics, 189
Hall. The Medical Examination for Life Assurance, 14
Holmes. Exercise for Health, 14
Hulbert and Phelan. Exercise for Health, 14
Jayne. Mammalian Anatomy, 207
Jewett. Essentials of Obstetrics, 65
Jones. Asepsis and Antisepsis in Abdominal Surgery and Gynæcology, 413
Jones. Llangammarch Wells as a Health Resort, 135
Klein. Elements of Histology, 447
Lahmann. Natural Hygiene, 431
Lefert. Lexique-Formulaire des Nouveautés Médicales, 153
Lippincott. *See Greene.*
Macdonald. Clinical Text Book of Surgical Diagnosis, 97
McVail. Report of the Royal Commission on Vaccination: A Review of the Dissentient Statement, 153
Manson. Tropical Diseases, 431
Martindale. The Extra Pharmacopœia, 431
Maw (S.), Son, and Thompson's Price List, 33
Monell. Manual of Static Electricity, 14
Monserrat. Surgical Technics in Hospital Practice, 311
Morris. Ringworm in the Light of Recent Research, 275
Moullin. Inflammation of the Bladder and Urinary Fever, 135
Newsholme. Origin and Spread of Pandemic Diphtheria, 393
Norris and Oliver. System of Diseases of the Eye, 65
Parkinson. Diagnosis of Disease, 117
Pearmain and Moor. Applied Bacteriology, 413
Prichard. Some Incidents of General Practice, 189
Pritchard. London and Londoners, 1898, 311
Ribot. Psychology of the Emotions, 207
Rideal. Practical Organic Chemistry, 447
Roth. Ethnological Studies among the North-West Queensland Aborigines, 33
Schofield. The Five Laws of Health, 447
Schwarzbach. Consumption: How to Avoid it and Weak Eyes, 189
Sladen (edited by). Who's Who, 1898, 153
Smith. Responsible or Irresponsible? 413
Snow. Twenty-two Years' Experience in the Treatment of Cancerous and other Tumours, 375
Sonnenschein. A Bibliography of Medicine, 33
Spencer. Outlines of Practical Surgery, 311
Stedman (edited by). Twentieth Century Practice of Medicine, 65, 375
Story. Transactions of the Royal Academy of Medicine in Ireland, 241
Tebb. A Century of Vaccination, and What it Teaches, 291
Thomson. Guide to the Clinical Examination and Treatment of Sick Children, 413
Twentieth Century Practice of Medicine, Vol. x., 65; Vol. xi., 375
Williams. Diseases of the Upper Respiratory Tract, 135
Brain and Nerves, Surgery of the (P.), 222
Branchial Carcinoma (P.), 115
Breasts, Cysts of the (P.), 64
Bristol Hospital for Sick Children, The (C.), 28
British Association, The, 420
British Laryngological, Rhinological, and Otological Association (C.), 129
British Medical Association, Edinburgh, 301
British Pharmacopœia, 1898, The (C.), 43
Bronchitis (P.), 371
Bullets, Explosive (C.), 129
Butter, The Purification of Bad, 331
Calculi, Prostatic (P.), 373
Calculus, Renal (P.), 290
Calculus, Vesical (P.), 255, 372
Cancer Mortality, Supposed increase of (P.), 95
Cancer, Oophorectomy in (C.), 202
Cancer of the Rectum (P.), 324
Cancer of the Stomach (P.), 411
Carcinoma, Branchial (P.), 115
Carcinoma of the Breast (P.), 95, 115
Carcinoma, Primary of the Pleura (P.), 95
Casualty Room Failures (W.), 378
Catheter Fever (C.), 427
Cerebral Surgery (P.), 328
Cerebral Tumours (P.), 239
Cervical Sympathetic, Neuro-fibromata of, 411
Chancres (P.), 373
Charred Straw as a Surgical Dressing (C.), 269
Chest, Surgery of the (P.), 184, 204
Children, Defective and Epileptic (W.), 360
Children, Diseases of (P.), 254
Children's Hospital, Melbourne, The (W.), 236
Cholelithiasis (P.), 11, 256
Chloroform Administration, 54
Chloroform Anæsthesia (C.), 220
Chondro-Osteosarcoma of the Breast (P.), 64
Circulatory System, Diseases of the (P.), 354, 428
Coats, Bags and Puerperal Fever, 264
Collins, "Dr.," The Case of, 247
Coloboma, Upward, of the Iris, Case of (P.), 13
Colotomy, Lumbar, Mr. Lawson Tait on, 253
Colour-hearing (P.), 237
Companies and Medical Practice (C.), 183
Compounding a Felony! 69
Conjunctivitis (P.), 12, 309
Conscientious Objector, The, 231
Conscription, 349
Consumption, Modern Views About, 178
Contemptible Fees, 365
Continental Hospital, A (W.), 450
Co-operative House Buying, 89
Co-operative Production, 109
Cornea, Diseases of the (P.), 46
Cornea, Tattooing of (P.), 12
Corneal Wounds (P.), *See p.* 94
County Council and Nuisances, The, 213
Coxa Vara Congenita (P.), 273
Cranial Surgery (P.), 204
Creosote (P.), 114
Crimino-Legal Responsibility of the Mentally Defective Psychologically Considered, The (C.), 368
Croydon Borough Hospital (W.), 342
"Cry of the Children, The," 283
Cystitis, The Bacteriology of (P.), 372
Cysts and Cystic Tumours of the Breasts (P.), 64
Cysts, Prepuce (P.), 373
Dacrocystitis, Etiology of (P.), 93
Death Certificates, The Law as to (C.), 76
Dentists, The Education of (C.), 236
Dermatology, Periscope of (P.), 151, 172
Dermato-Neuroses (P.), 173
Diabetes, On the Treatment of (C.), 236
Diagnosis, An Erroneous (C.), 61
Diet and Suicide, 331
Digestive Organs, Diseases of the (P.), 11, 29, 410, 443
Diphtheria, Heart Complication in (P.), 354
Diploma, Choice of a, 385
Disinfection of Excreta, The (C.), 60
Drink and Insanity, 333
Dropsy (P), 130
Dropsy, The Meaning and Treatment of (C.), 253
Drowned in Dust (C.), 442
Drunkenness among Women, The Cause of, 270
Ear. Complications of Middle Ear Disease, 204
Ear Replaced after Complete Severance (C.), 183
Easily Satisfied (C.), 409
East Finchley Convalescent Home (W.), 98. *See p.* 122
Economics of Philanthropy (W.), 66, 118, 136, 191
Eczema (P.), 152
Electrical Hospital? An (W.), 50
Electrical Hospital, A Free, 39
Edinburgh Meeting, The, 297
Edinburgh Meeting, Addresses, 301
Endocarditis, Acute Inflammatory Diseases of, 354
Endocarditis (P.), 113, 428
Endometritis, Chronic, Bacteriology of, 339
English and German Midwifery Teaching, 163
Entropion, Operation for (P.), 310
Enucleation or Hysterectomy (C.), 76
Epidemic of Suspicion, An, 299
Epilepsy (P.), 239
Epithelioma of the Face (P.), 116
Eserin in Corneal Ulcer (P.), 46
Eucalyptus in Actinomycosis (C.), 169
Exophthalmic Goitre (P.), 63
Expert Witness, The, 421
Eye Weariness (C.), 235
Factory Acts, Breaches of the, 281
Feeble-minded Children, 3
Feet, The Care of the (C.), 269
Fevers (P.), 337, 353, 370, 390
Fibroid Tumours of the Uterus (C.), 234
Fibro-Sarcoma of the Sciatic Nerve (P.), 64
Flash-point of Petroleum, The, 55
Flora of Healing Wounds (C.), 10
Foreign Bodies in the Eye (Localisation of by the Röntgen Rays) (P.), 46
Foreign Bodies left in the Abdomen (C.), 112
Fractures and Dislocations (P), 149, 171, 429
Fresh Air for City Children, 437
Ganglion, Simple and Compound, On (C.), 42, 58
Gas Lighting, A Revolution in (W.), 193

Gastric Ulcer (P.), 134

Gastro-Jejunostomy (C.), 442

Gastroptosis (P.), 443

"Gears," The Question of, 402

General Surgery (P.), 116, 149, 171, 412, 429, 444

Genito-urinary Surgery (P.), 30, 45, 289, 308, 372

Genu Recurvatum (P.), 223

Genu Valgum, The Pathology of (P.), 402

Glaucoma, Mydriatics in (P.), 310

Goitre, A Clinical Lecture on (C.), 146, 165, 182

Goitre, Exophthalmic (P.), 63

Gonorrhœa, Eye Complications in (C.), 90

Gosforth Home for Crippled Children (W.), 242

Gout, The Alkalinity of the Blood in (C.), 170

Gout, The Treatment of, by Alkalies (C.), 202

Gout, Rheumatism and (P.), 61, 77

Gout, Vegetables and (C.), 219

Guaiacol in Phthisis (C.), 44

Gynæcology, American Comment on London, 317

Hæmato-Myelia from Gunshot Wounds of the Spine (P.), 373

Hæmorrhoids (P.), 324

Hair Washes, Inflammable, 437

"Hard Case," A, 179

Harrogate Water, The Action of (C.), 233

Head Injuries, Inflammatory Sequelæ of (P.), 222

Health Resorts, Cautions as to (C.), 201

Heart, Acute Dilatation of the (C.), 254

Heart and Circulation, Diseases of the (P.), 62, 79, 92, 113, 130

Heart Disease (P.), 323

Heart Disease, Exercises in, 43, 355

Heart Disease, Iodides in Treatment of (P.), 130

Heart Disease, Nauheim Treatment of (C.), 352

Heart, Effect of Pregnancy on the (P.), 428

Heart, Acute Inflammatory Diseases of the, 354

Heart, Modes of Death in Punctured Wounds of the (C.), 427

Heart, Relaxation of the (P.), 354

Heart, The Treatment of Acute and Inflammatory Diseases of the (C.), 28

Heart, Tropical (C.), 389

Henoch's Purpura (P.), 77

Hepatic Abscess (P.), 257

Hepatoptosis, or Floating Liver (P.), 257

Hernia (P.), 31, 340, 355

Hernia, Infantile Umbilical (C.), 128

Hernia of the Linea Alba (P.), 29

Hernia of the Lungs (P.), 204

Hernia, Radical Cure of (P.), 356

Hernial Sac, Tuberculosis of the (P.), 355

Hip-joint Disease, Deformity Arising from (C.), 7

Holocaine (C.), 409

Home Treatment of Disease, On the (C.), 321

Homestead Law, 127

Hospital Administration (W.), 261, 276, 450

Hospital Bazaar, The, 231

Hospital Construction (W.), 98, 242, 342, 414

Hospital Festivals, Meetings, &c., 19, 34, 49, 67, 82, 101, 119, 137, 154, 174, 192, 209, 226, 243, 262, 277, 294

Hospital Fittings, 15

Hospital Housekeeping (W.), 358, 377, 398

Hospital, A Military, 100 Years Ago, 399

Hospital Sunday Fund, The (W.), 451

Hospitals and Accidents, 215

Hospitals, Complaints against, 364

Hospitals (Other) at which Clinical Instruction is Given, 397

Hot Weather Precautions, 365

Hybrids, 87

Hydatid of Brain (P.), 239

Hydatids, Abdominal (P.), 221

Hydrocephalus, Drainage for (P.), 239

Hydrocephalus, Surgical Treatment of (P.), 255

Hydrogen Dioxide in Marginal Blepharitis, 310

Hydronephrosis (P.), 30

Hygiene in the East and in the West, 214

Hyoscin and Hyoscamin as Mydriatics (P.), 94

Hyper-acidity, Paroxysmal (P.), 255

Hypnotism, 281

Hypnotism and Crime, 419

Hysterectomy, Abdominal, for Fibroids (P.), 32

Hysterectomy, Vaginal (P.), 32

Ice Creams Again! 197

Icthyol (P.), 114

Imbecility (P.), 255

Inebriates Bill, The, 71, 165, and *see p.* 211

Infanticide, The Judicial Treatment of, 181

Infantile Paralysis (C.), 9

Infectious Diseases in General Hospitals, 317

Influence of One Disease upon Another (C.), 111

Inorganic Constituents of Vegetable Food, 348

Inquests and Uncertified Deaths, 71

Insane, The After-Care of the, 179

Insane, The Story of the Insane from Year to Year (W.), 68, 139

Insanity, Hints for the Treatment of Suddenly-Developed (C.), 200, 218

Insanity, The Plea of, 330

Insanity, Prognosis in (C.), 284, 304

Insurance, The Selection of Lives for (C.), 320

Intrapleural Tension (P.), 410

Intussusception, A Case of (C.), 335

Iodides in Treatment of Heart Disease (P.), 130

Iodin in Trachoma (P.), 310

Iodoform Gauze in Ruptured Uterus (C.), 286

Iritis after Slight Corneal Injuries (C.), 93

Juvenile Reformatories, 145

Keratitis, Interstitial (P.), 12

Kidney, Floating (P.), 31

Kidney, Movable (P.), 290

Kidney, Sarcoma of (P.), 30

Labour in Mature Primiparæ (P.), 48

Labour, The Obstruction of (P.), 48

Ladies and Tobacco, 85

Lameness, Intermittent (P.), 429

Lamp Accidents, The Causes of, 317

Laryngeal Stridor (C.), 10

Lead as an Abortifacient (C.), 321

Lead Poisoning Among Compositors (C.), 409

Lead in the Potteries, 197

Legislation and Public Health, 329

Leper Hospitals of the West Indies, The (W.), 244, 260, 292

Leprosy (P.), 151

Leucocytes, The Physiological Role of, in Corneal Wounds (P.), 94

Lipomata (P.), 64

Litten's Sign (P.), 221

Liver, Accessory Lobe of (P.), 257

Liver, Floating (P.), 257

Liver, Hydatids of (P.), 257

Liver, Operations on the (C.), 6, 74

Liver, Traumatic Rupture of the (P.), 256

Liver, Tumours of the (P.), 258

Liverpool, The Health of, 215

Lock Stitch in Surgery, The (C.), 286

Locomotor Ataxy (P.), 238

London Air, The Actinic Opacity of, 316

London Smoke, 141

London University, The, 196

Long Bones, Operations upon the (P.), 223

Lower Extremity (General Surgery) (P.), 444

Lung, Hernia of the (P.), 204

Lungs, Diseases of the (P.), 371, 409

Lupus (P.), 172

Lying-in Institutions, Private, 23

Lymph, Preparation of Glycerinated Calf, 147

Mal-practice, Action for (C.), 112

Malpraxis, 248

Mansion House Dinner, The, 105

Marginal Blepharitis, Treatment (P.), 310

Marriage of First Cousins, The, 421

Marriage of the Unfit (C.), 426

Mastoid Disease (P.), 204

"Matching" of Milk, The, 107

Measles, Isolation, after, 125

Measles and Whooping Cough, Control of (C.), 91

Meat, The Inspection of, 265

Meat, The Traffic in Bad, 163

Medical Aid in Time of War (W.), 448

Medical Attendance upon Servants (C.), 92

Medical Council, The (C.), 167

Medical Council and Midwives, The, 38

Medical Degrees and Diplomas, 397

Medical Education and Charity, 53

Medical Ethics, 143

Medical History, an Episode in, 303

Medical Holidays, 401

Medical Missionaries, 249

Medical Missions, 363

Medical Mrs. Grundy, A (C.), 219

Medical Orthodoxy in the Far East, 162

Medical Practice, Companies and (C.), 183

Medical School, Choice of a, 386

Medical School, Entry at a, 387

Medical Schools, 394, 396, 397

Medical, Surgical, and Hygienic Exhibition (W.), 191, 208

Medical Women, 251

Medieval Remedies for the Plague, 199, 217, 233

Menière's Disease (P.), 238

Meningitis (P.), 271

Meningocele and Spina Bifida (P.), 185

Mesenteric Lymph Glands, Tuberculosis of, 222

Metropolitan Asylums Board, 226, 249, 299

Metropolitan Hospital Sunday Fund, 343, 376

Metropolitan Press and the Hospitals, The, 21

Microcephaly, Surgical Treatment of (P.), 255

Middle Class Hospitals, 39

Middle Ear Disease, Complications of (P.), 204

Middlesborough Epidemic, The (C.), 76

Middlesex Hospital, The Story of the (W.), 361

Midwifery, Charity, in London, 436

Midwives' Bill, The, 107, and *see p.* 121

Midwives' Registration Bill, The, 27

Migraine (P.), 238

Mitral Stenosis (P.), 323

Modern Operating Theatres (W.), 312

Modern Sociology, 5, 25, 41, 57, 73, 89, 109, 127, 145, 165, 181, 199, 217, 233, 251, 267, 283, 333, 367, 405, 423, 439

Molluscum Contagiosum, (C.), 60

Morphomania (C.), 8

Mortality in War, 179

Mother in the Factory, The, 5

Movable Kidney (P.), 290

Munyon's "Remedies," 161

Mydriatics, Hyoscin and Hyoscamin as (P.), 94

Myomectomy, Abdominal. *See p.* 339

Nephrectomy (P.), 45

Nephrectomy, Limits of (P.), 290

Nephritis, Acute, in Children (P.), 287

Nephropexy (P.), 45

Nephro-uretectomy (P.), 45

Nerve, Inferior Dental, Removal of the, through the Mouth (P.), 411

Nerves (and Brains), Surgery of the (P.), 223, 411

Nerves, Peripheral, Course and Treatment of Injuries to the (P.), 411

Neuralgia, Osmic Acid in (C.), 44

Neuralgia, Paræsthetica (P.), 289

Neuritis, Multiple (P.), 288

Neurology, Progress in (P.), 237, 256, 271, 288

Neurotics and Cardio-Vascular Neuroses (C.), 10

New Appliances and Things Medical, 13, 32, 48, 96, 116, 152, 173, 189, 206, 224, 240, 258, 274, 290, 326, 374, 392, 412, 446

New Growths (P.), 64, 95

Notes from Australia (W.), 226

Notes and News, 4, 24, 40, 56, 72, 88, 108, 126, 144, 164, 180, 198, 216, 232, 250, 266, 282, 300, 318, 332, 350, 366, 384, 404, 422, 438

Notification of Isolation (C.), 335

Nurses for Board Schools, 279

Nursing in Prison Infirmaries 174, and *see p.* 194

Obstetrics and Gynæcology, Progress in (P.), 31, 47, 148, 325, 338

Occupational Mortality (P.), 356

Œsophageal Pouch (C.), 44

Œsophago-Enterostomy (P.), 133

Œsophagus, "Pressure Pouches" of the (P.), 133

Oophorectomy in Cancer (C.), 202

"Open Air," What is Meant by? (C), 306, 328

Operating Theatres, Modern (W.), 312

Ophthalmology, Progress in (P.), 12, 46, 93, 308

Ophthalmoscopic Evidence of General Arterial Disease (P.), 46

Orthopædic Surgery (P.), 223, 273

Osmic Acid in Neuralgia (C.), 44

Osteoclasis, Manual (C.), 407

Osteostomy (C.), 407

Ovarian Cyst, Post-Typhoid Suppuration, 149

Ovariotomy, The Danger of Pregnancy after (P.), 148

Ovariotomy, Results of (P.), 149

Oxygen, The Use of, by Aeronauts, 437

Pancreas and Spleen, Surgery of the (P.), 239

Paræsthesia (P.), 289

Paralysis of the Arm (P.), 288

Paralysis, Facial (P.), 288

Paraphlegia, Hereditary Spastic (P.), 271

Paris and London Water, 215

Patella, Fracture of the (C.), 285, 425

Pauper Sick in London, The (W.), 416

Payment for Notification (C.), 44, and *see p.* 84

People's Banks, 423

Peptone (P.), 307

Pericarditis (P.), 79

Peritoneum, Surgery of the (P.), 205, 221

Petroleum, The Flash Point of, 299

Philanthropy. *See Economics of Philanthropy*

Phimosis (P.), 373

Phosphorus Poisoning, The Diagnosis of, 197

Phthisis and Compression of the Lung (C.), 268

Phthisis, Infection in (P.), 132

Phthisis, Open-air Treatment of (P.) 132 (C.) 147

Phthisis, Outdoor Treatment of, at Cromer, 8

Phthisis Tuberculin (P.), 114

Physician in Perplexity, The, 347

Physician, The Title of (C.), 220

Physicians, Felonious (C.), 254

Placenta, Prolonged Retention of the (C.), 442

Plague, The, 23 (P.), 391

Plague, Mediæval Remedies for the, 199, 217

Pleural Effusions (P.), 184

Pleurisy (P.), 409

Pleuro-Pneumonia, 125

Plymouth Borough Hospital (W.), 451

Pneumonia (P.), 203, 271

Pneumonia and Broncho-Pneumonia (C.), 169

Pneumonia, Diphtheritic (P.), 371

Pneumonia after Injury (P.), 372

Pneumonia, Streptococcic (P.), 372

Pneumonia, Treatment of, by Salicylic Acid (P.), 372

Pneumo-Thorax (P.), 204

Poor Law Sanatorium, A, 437

Population Question, The, 3

Post-Graduate College for London, A, 142

Potts' Disease and its Results (P.), 186

Prepuce Cysts (P.), 373

Press Bazaar in Aid of the London Hospital, 243

Prince of Wales's Hospital Fund, The (W.), 432

Prison Administration and the Training of Officials, 25, 41, 73

Prison Reform, 57

Prisoners, The Treatment of. *See p.* 104

Private Fees in Public Hospitals (W.), 99, 121

Privilege, A Question of, 28

Profession, Choice of a, 381

Prostate (P.), 309

Prostate, Enlarged (P.), 373

Prostatic Calculi (P.), 373

Provident Dispensaries, 403

Public Bodies and Workmen's Dwellings, 312

Pulmonary Abscess (P.), 204

Purpura and Allied Diseases (P.), 177

Pylorectomy (P.), 133

Pyloric Stenosis (P.), 134

Pyo-Pneumothorax (P.), 204

Quacks, The Prosecution of, 382

Queen Charlotte's Lying-in Hospital, 433

Queen's Jubilee Hospital, The (W.), 80

Rabies (P.), 390

Railway By-Laws, 195

Rate-Supported Hospitals, 123

Reception House in St. George's, Southwark, 87

Rectal Aspiration (P.), 324

Rectal Stricture (P.), 324

Rectum and Anus (P.), 324

Rectum, Cancer of the (P.), 324

Rectum, A Pin in the, for Thirty Years (C.), 442

Rectum, Primary Tuberculosis of the (P.), 324

Reduplication of Sounds (P.), 323

Renal Calculus (C.), 183, 290

Renal Disease, The Blood in (P.), 30

Renal Medicine (P.), 287, 306

Renal Pelvis and Ureters, Surgery of (P.), 308

Renal Surgery (P.), 391

Rescue Work, Methods of, 267

Respiratory Centre, Paralysis of the (P.), 222

Respiratory Organs, Diseases of (P.), 114, 13', 202, 221

Retrotarsal Fold, Large Fragment of Wood in (P.), 309

Rheumatism and Gout (P.), 61, 79

Rheumatoid Arthritis (P.), 78

Rickety Curvature of the Legs, 406

Rinderpest, Inoculation against (C.), 369

Röntgen Rays, Examination by (P.), 79

Röntgen Rays, Use of in Diagnosis (P.), 355

Ropner Convalescent Home, *See* p. 295

Royal College of Surgeons of England (C.), 60

St. Neots Murder, The, 177

Salicylic Ointment in Spring Catarrh (P.), 12

Sanitary Authorities and Dangerous Wells, 55

Sanitary Service, The 37

Saprophytic Pneumococci (C.), 91

Sarcoma of Kidney (P.), 50

Sawdust Urinals (C.), 428

Scarlet Fever, The Causes of Prolonged Infectivity in (C.), 407

Scarlet Fever, Return Cases of (C.), 441

School or Barrack? 107

School Boards and Medical Certificates (C.), 59

Sciatic Nerve, Fibro-sarcoma of the (P.), 64

Science, The Teaching of Elementary (C.), 147

Sclerosis, Multiple (P.), 254

Seasonable Variation in Disease (C.), 219

Secondary Education, 229

Serum, Anti-streptococcic (C.), 61

Sewage and Refuse Disposal (P.), 186

Shell Wounds in the American Navy, 184

Skull, Depressed Fracture of the, The Question of Operating in (P.), 223

Small-pox (P.), 370

Small-pox Hospital, Barnsley (W.), 342

Small-pox Hospital, a Nuisance, Is a? 49

Small-pox, The Re-introduction of, 367, 405

Snake Poisons (P.), 390

Spina Bifida, Meningocele and (P.), 185

Spine, Non-Tubercular Affections of (P.), 185

Spine, Surgery of the (P.), 373

Spleen and Pancreas, Surgery of the (P.), 239

Splenectomy (P.), 239

Spondylolisthesis (P.), 273

Statutory Holidays, 143

Stenosis, Mitral (P.), 323

Stenosis, Pyloric (P.), 134

Sterilisation by Formaldehyde, (C.), 77

Stomach, Cancer, Syphilis and Tuberculosis of the (P.), 411

Stomach, Dilatation of the (P.), 443

Stomach, Hour-glass Contraction of the, 134

Stomach, Spasmodic Stricture of (P.), 443

Stomach, Total Extirpation of the (C.), 286

Street Noises, 124

Stricture, Rectal (P.), 324

Student of Medicine, The, 52, 68, 140, 160, 228 246, 314, 328, 362, 380, 417, 434

Stuffy Houses, 143

Suburban Villas and Gravel Soil, 383

Suicide by Gas, 55

Surgery, The Present Position of, 302

Syphilis (P.), 373

Syphilis, Some Forms of Tertiary (C.), 252

Syphilis of the Stomach (P.), 411

Tabes, The Treatment of (C.), 143, (P.), 256

Technical Education, 383

Technique: A Word to Students, 70

Teetotallers, The Sins of, 298

Tenon's Capsule, Insertion of Artificial Globe into (P.), 12

Test of a Good Citizen, The, 265

Tetanus (P.), 390

Tetany (P.), 255

Therapeutic Notes (C.), 170, 352

Tic (P.), 289

Tobacco, A Whiff of, 231

Tonsil in Tuberculosis, The (C.), 234

Toxic Materials released from Fæces, (C.), 388

Trachoma, Iodin in (P.), 310

Trephining for Apoplexy (C.), 170

Trephining for Idiocy (P.), 239

Tropical Diseases, A School for the Study of, 263

Tropical Heart (C.), 389

Tropical Hygiene, 86

Tuberculin, Phthisis (P.), 114

Tuberculosis of the Bladder (P.), 373

Tuberculosis in Cold-blooded Animals (C.), 59

Tuberculosis Commission, The, 71

Tuberculosis, The Crusade Against, 281

Tuberculosis of the Hernial Sac (P.), 355

Tuberculosis, The Open-Air Treatment of, 328

Tuberculosis, Renal (P.), 45

Tuberculosis of the Stomach (P.), 411

Tuberculosis, The Tonsil in (C.), 234

Tuberculous Disease of the Bladder (C.), 130

Tumours, Special (P.), 64

Tumours, The Treatment of Inoperable (P.), 95

Typhoid (P.), 337, 353

Typhoid, Abnormal (C.), 322

Typhoid Fever, The Antiseptic Treatment of, 130

Typhoid, The Victims of, 349

Typhoid Fever, Perforation in Successful Operation (C.), 305

Typhoid, Widal's Test for (C.), 408

Typhus, A Nursery of, 262

Typography and Eye-Weariness (C.), 235

Ulcer, Gastric (P.), 134

Ulcer of the Stomach (P.), 410

Underfed Children, 87

Unqualified Assistant (C.), 201

Unqualified Practice, The Legality of (C.), 270

Uræmia (P.), 287, 336

Ureters, Surgery of the (P.), 4[illegible], 308

Urethra, "Splicing" the (C.), 76

Urethra, Stricture of the (P.), 45

Urethral Calculi (P.), 373

Urinary Affections, Surgical, in Children (P.), 45

Urine, Toxicity of (P.), 307

Uterus, Cancer of, Pregnancy with, (P.), 47

Uterus, Inversion of the (P.), 325

Uterus, Iodoform Gauze in Ruptured (C.), 286

Uterus, Rupture of the (P.), 325, 338

Uterus, Prolapsed, and Vagina, Acute Strangulation of (P.), 339

Vaccination Bill, The, 315

Vaccination, Compulsory (C.), 306

Vaccination, Deaths from, 3

Vaccination by Private Practitioners, 22

Vaginal Cœliotomy for Removal of Multiple Uterine Myomata (P.), 47

Variola and Vaccinia, Relationship Between, 106

Vegetables and Gout (C.), 219

Venereal Diseases (P.), 309

Vermiform Appendix, Surgery of (P.), 110, 272

Vesical Irrigation (P.), 372

Virulence and Non-Virulence (C.), 235

Visitors, Lady, to Her Majesty's Prisons, 439

War and Disease, 125

War in Hot Countries, 403

Water Companies and Sanitary Authorities, 265

Water Supplies, The Control of Public, 71

Water Supply in East London, The, 383

Water Supply and Public Health, 403

Water, The Waste of (C.), 426

Wedge, Removal of a, from the Bone (C.), 407

Whooping Cough and Measles, Control of, 91

Women and Dangerous Trades, 281

Women Inspectors under the Factory Acts (C.), 306

Word to Hospital Critics, A, 1

Workmen's Compensation Act, The (C.), 76, 249

Wounded, Laws of War relating to the (W.), 159

Wounds by Small Projectiles (C.), 129

Xerostomia (P.), 410

Yellow Atrophy, Acute (P.), 11

Yellow Fever (P.), 391

### THE SPECIAL SUPPLEMENT FOR NURSES.

American Letter, Our, 55, 94, 125, 151, 190
American Red Cross Society, 206
American Women and War, 48
Antiseptics for Nurses, 3, 21, 27, 123, 139, 155, 163, 171, 179, 187, 195, 212
Appointments, 33, 39, 49, 57, 66, 82, 95, 100, 111, 127, 134, 142, 149, 157, 174, 181, 197, 206, 222
Appointments, Minor, 11, 24, 33, 49, 55, 68, 76, 82, 95, 102, 111, 117, 124, 134, 142, 151, 159, 167, 175, 183, 190, 197, 206, 214, 222
Association of Asylum Workers, The, 5
Bangor Hospital Enquiry, The, 118
Bishop Auckland and District Nursing Association, 94
Bombay, A Letter from, 63, 173
Book and Its Story, A, 10, 31, 47, 75, 95, 103, 150, 166, 180, 198, 221
Book World for Women and Nurses, The, 15, 24, 40, 63, 127, 150, 168, 175
British National Red Cross Society, The, 193
Captive Sisters at Khartoum, The, 204
Care of the Feeble-minded, 109
Carnival in a Children's Hospital, 32
Central Bureau for the Employment of Women, 84
Charming Holiday Resort, A, 183
Concert at Stafford House, Grand, 67
Cottage Hospital and Nurses' Home at Acton, New, 57
County Nursing Association, How to Start a, 113
Cremation, 11
Death in Our Ranks, 10, 23, 34, 54, 65, 118, 133, 167, 182, 199, 222
Diet for Invalids, 8
Duel in Germany, The, 77
Epileptic Colony, Chalfont St. Peter, The, 174
Everybody's Opinion, 15, 24, 33, 41, 56, 67, 74, 87, 103, 110, 119, 127, 135, 143, 151, 159, 167, 174, 183, 191, 199, 207, 223
Florence Nightingale on Nursing, 117
Grosvenor Hospital for Women and Children, 174
Guy's Hospital Nurses and Students' Concert, 74
Gynæcological Nursing, Notes on, 132, 147, 211
Hamilton, M.D., Miss Lilias, at the Royal British Nurses' Association, 12
Hobbies and Accomplishments, 72
Hotel for Nurses, The, 24
Hygiene made Popular, 86
In Memoriam, 110
Inniskea Nurses, Investiture of the, 197
International Fancy Fair at Camden Town, The, 65
Japan, 135
Lecture to Nurses, 71, 91, 99
Lecture to Surgical Nurses, 19, 29, 45, 53, 61, 81
Life of an Incurable, The, 205
London School of Medicine for Women, 143
Matron's Council, The, 111, 118
Meath Home of Comfort for Epileptics, 159
Meath Workhouse Attendants' Association, The, 64
Mediæval Practice in the Care of the Sick, 14, 64
Medical Women and Nursing Sisters, 167
Milkmaid Nurse: An Irish Jumble, The, 175, 199
News from the Nursing World, 1, 17, 27, 35, 43, 51, 59, 69, 79, 89, 97, 105, 113, 121, 129, 137, 145, 153, 161, 169, 177, 185, 193, 201, 209, 217
Nightingale (Florence) on Nursing, 117
Notes on Gynæcological Nursing, 132, 147, 211
Notes and Queries, 16, 26, 34, 42, 50, 58, 68, 78, 88, 96, 104, 112, 120, 128, 136, 144, 152, 160, 168, 176, 184, 192, 200, 208, 216, 224
Novelties for Nurses, 6, 37, 86, 100, 117, 157
Nurses for Board Schools, 49
Nurses' Home at Marylebone Infirmary, New, 65
Nurses and Note-Books, 20
Nursing Arrangements at the Victoria Park Hospital, 148
Nursing Developments in London Institutions, 5
Nursing in Diseases of the Throat, Nose, and Ear, 148, 203, 219
Nursing in India, 111, 133
Nursing of Mediterranean Fever, The, 39
Nursing in Paris Hospitals, 7, 25, 30, 38, 62, 73, 83, 93, 101, 108, 124, 164, 189
Nursing Sisters of St. John the Divine, The, 12
Nursing in Workhouses, 72
Parents' National Educational Union, 76, 85
Physical Exercises at the Queen's Hall, 109
Plaistow Nurses Again, The, 195
Poonah, A Letter from, 13
Post-graduate Clinics for Nurses, 4, 23, 46, 54, 66, 82, 92, 107, 115, 131, 172
Presentations, 24, 33, 39, 49, 56, 62, 73, 87, 108, 115, 127, 139, 147, 160, 173, 182, 197, 216, 222
Press Bazaar, The, 133
Prevention of Cruelty to Children, The Society for the, 119
Pro's Resolve, A, 190
Promotion, 13
Qualifications of a Really Good Nurse, Some, 182
Queen Charlotte's Hospital, 134
Queen's Nurses at Grosvenor House, The, 142
Queen Victoria's Jubilee Institute for Nurses, 125
Reading to the Sick, For, 26, 34, 42, 50, 58, 68, 78, 88, 96, 104, 112, 120, 128, 136, 144, 152, 160, 168, 176, 184, 192, 200, 208, 216
Reply to "A Sister's Lament," The Nurse's, 232
Responsibility for Injury Sustained on Duty, 33
Royal British Nurses' Association, 5, 86, 123, 140
Royal British Nurses' Association, Miss Lilias Hamilton, M.D., at the, 12
Royal National Pension Fund for Nurses, 214
St. Luke's Home, Vancouver, B.C., 190
Sholapore Plague Hospital, 207
Sick Diet: Milk, 22
Sister Ellen, of the Walsall Hospital, 199
Sister's Lament, A, 174
Society for the Prevention of Cruelty to Children, The, 119
Soldier's Nurse, The, 181
State Children's Aid Association, 55
State Registration for Nurses, Actual and Possible Benefits of, 56
Sweden, Trained Nursing in, 9
THE HOSPITAL Convalescent Fund, 134, 157, 197, 211
Training in the Provinces, 116, 126, 141, 156, 165, 181, 188, 205, 215, 220
Walk With a Weinyddes (Nurse) of Wild Wales, A, 214
Walsall and District Hospital, 5
Wants and Workers, 26, 49, 78, 85, 144, 148, 168, 180, 188, 197, 205, 222
Where to Go, 34, 37, 65, 77, 87, 96, 102, 115, 125, 134, 140
Woman's Hospital in Morocco, A, 125
Worcester General Infirmary, 40
Workhouse Infirmary Nursing Association, 85
Work and Workers in India, 149
Zenana Bible and Medical Mission, The, 43
Zenana Bible and Medical Mission, Meetings on Behalf of the, 55

Printed by W. H. AND L. COLLINGRIDGE, City Press, 148 and 149, Aldersgate Street, London, E.C.

IMPORTANT NOTICE.

On account of the Easter Holidays, we beg to announce that we shall go to press with our issue of the 9th APRIL on TUESDAY NIGHT, the 5th INST. Advertisements intended for insertion in this issue, should therefore reach us BY NOON ON TUESDAY, the 5th inst.

Notices of Births, Deaths, and Marriages are inserted in this column at a charge of 2s. 6d. for an announcement not exceeding four lines.

### NOTICE TO CORRESPONDENTS.

All MS., Letters, Books for Review, and other matters intended for the Editor should be addressed The Editor, "The Hospital," The Hospital Building, 28 & 29, Southampton Street, Strand, W.C.

The Editor cannot undertake to return rejected MS., even when accompanied by stamped directed envelope.

NOTICE.—Vol. XXIII. of "The Hospital" (October, 1897, to March, 1898), in handsome cloth binding, is now on sale at the Office of this Journal, price 7s. 6d. Cases for binding the Numbers contained in this Volume, 1s. 6d. Index to Vol. XXIII., 2½d., post free.

The Monthly Part for April is now ready. Price 6d., by post 8d.

### Notes and News.

### THE STUDENT OF MEDICINE.

### APPOINTMENTS.

E. W. Adams, M.B.Lond., appointed Resident House Physician to the Sheffield Royal Hospital. P. D. Barker, M.R.C.S., L.R.C.P., appointed Junior House Surgeon to the Chesterfield and North Derbyshire Hospital and Dispensary. H. R. Brown, M.B., C.M.Edin., appointed Medical Officer of Health to the Maldon Urban Sanitary District. W. S. Church, M.D.Oxon, F.R.C.P.Lond., appointed a Consulting Physician to the Royal General Dispensary, Bartholomew Close. E. F. Clowes, L.R.C.P.Lond., M.R.C.S.Eng., appointed House Physician to the Royal Hants County Hospital, Winchester. Sir D. Duckworth, M.D.Edin., F.R.C.P.Lond., appointed a Consulting Physician to the Royal General Dispensary, Bartholomew Close. W. E. Fairweather, M.R.C.S.Eng., L.R.C.P.Lond., appointed Assistant House Surgeon to the Rotherham Hospital. Dr. Groves, appointed Medical Officer for the Chewton Mendip District of the Wells Union. F. Hawthorn, M.B., B.S., M.R.C.S., L.R.C.P., appointed Teacher and Examiner of Vaccination at Newcastle-on-Tyne Educational Vaccination Station. G. A. Jelly, M.R.C.S., L.R.C.P., L.S.A., appointed House Surgeon to the Sunderland and North Durham Eye Infirmary. T. C. Lawson, M.R.C.S.Eng., L.S.A., appointed Medical Officer for the Fourth District of the Devizes Union. T. Mulock-Bentley, L.R.C.P., L.R.C.S.I., appointed by His Honour the President of the Free State Government District Surgeon for the District of Vrede. E. Pennington, M.R.C.S., L.R.C.P., appointed Medical Officer of Health for the No. 8 (Preston Candover) District of the Basingstoke Union. J. F. Porter, B.A.Dub., M.D., M.R.C.S., appointed Medical Officer for the combined District of Helmsley and Oswaldkirk of the Helmsley Rural District Council. J. E. P. Shera, L.R.C.S.I., L.R.C.P.I., L.M.Rotunda, appointed House Surgeon to the Teignmouth Hospital, South Devon. Sir T. Smith, Bart., F.R.C.S.Eng., appointed Consulting Surgeon to the Royal General Dispensary, Bartholomew Close. E. J. Smyth, M.D.Lond., B.S., appointed Medical Officer for the No. 4 District of the Buckingham Palace Workhouse of the St. George's Union. M. H. Way, M.R.C.S.Eng., L.R.C.P.Lond., appointed House Surgeon to Guy's Hospital. H. H. Woods, M.R.C.S.Eng., L.R.C.P.Lond., D.P.H.Cantab, appointed Medical Officer of the Milton District of Lymington Union.

